# Recycled Jute Non-Woven Material Coated with Polyaniline/TiO_2_ Nanocomposite for Removal of Heavy Metal Ions from Water

**DOI:** 10.3390/molecules29184366

**Published:** 2024-09-14

**Authors:** Aleksandar Kovačević, Marija Radoičić, Darka Marković, Zoran Šaponjić, Maja Radetić

**Affiliations:** 1 Faculty of Technology and Metallurgy, University of Belgrade, 11000 Belgrade, Serbia; akovacevic@tmf.bg.ac.rs; 2 “Vinča” Institute of Nuclear Sciences, University of Belgrade, 11000 Belgrade, Serbia; mradoicic@vinca.rs; 3 Innovation Centre of the Faculty of Technology and Metallurgy, University of Belgrade, 11000 Belgrade, Serbia; darka@tmf.bg.ac.rs; 4 Institute of General and Physical Chemistry, University of Belgrade, 11000 Belgrade, Serbia; zsaponjic@iofh.bg.ac.rs

**Keywords:** jute, recycling, sorbent, polyaniline, heavy metal ions

## Abstract

Growing volumes of textile waste and heavy metal pollution of water are emerging environmental challenges. In an attempt to tackle these issues, a non-woven sorbent based on jute fibers was fabricated by recycling the textile waste from the carpet industry. The influence of contact time, concentration, pH and temperature on the sorption of lead and copper ions from aqueous solutions was studied. In order to enhance the sorption capacity of the non-woven material, in situ synthesis of polyaniline (PANI) in the presence of TiO_2_ nanostructures was performed. The contribution of TiO_2_ nanoparticles and TiO_2_ nanotubes to the uniformity of PANI coating and overall sorption behavior was compared. Electrokinetic measurements indicated increased swelling of modified fibers. FTIR and Raman spectroscopy revealed the formation of the emeraldine base form of PANI. FESEM confirmed the creation of the uniform nanocomposite coating over jute fibers. The modification with PANI/TiO_2_ nanocomposite resulted in a more than 3-fold greater sorption capacity of the material for lead ions, and a 2-fold greater absorption capacity for copper ions independently of applied TiO_2_ nanostructure. The participation of both TiO_2_ nanostructures in PANI synthesis resulted in excellent cover of jute fibers, but the form of TiO_2_ had a negligible effect on metal ion uptake.

## 1. Introduction

Textile fiber production nearly doubled in the last 20 years due to population growth and improved living standards. Globally accepted fast fashion trends resulted in the accumulation of huge amounts of post-consumer textile waste that is either incinerated or sent to landfills (approximately 80%), with only 1% being recycled into new clothes [1]. Most of the EU directives are addressed to sustainable waste management practices for post-consumer textile waste [2]. Less attention has been paid to large volumes of post-industrial textile waste. Managing post-industrial textile waste is a serious issue particularly in developing countries where textile goods are mainly manufactured [3]. Although recycling is the preferable solution, this waste is more commonly incinerated or disposed in landfills. The worst practice is illegal waste dumping, which has a detrimental effect on the environment and poses a health risk to local communities. 

Another global concern is related to the presence of heavy metal ions in water. High concentrations of metal ions pose a threat to human health, and consuming drinking water contaminated with heavy metals can lead to various health issues, including cardiovascular disorders, neuronal damage, kidney damage, and an increased risk of cancer and diabetes [4]. Everyday exposure to heavy metal ions is common, for instance, galvanized pipes in water distribution systems release mostly zinc, followed by cadmium and lead. Other dangerous source of heavy metal ions (in particular Pb^2+^, Cu^2+^, Hg^2+^, Cd^2+^ ions) is wastewater from mining and ore processing, petroleum refining, metal plating, ceramics, fertilizer, pesticide, dyes and batteries production, the textile industry, etc. [5]. These also result in adverse effects to animals and plants when entering the environment in high concentrations.

Several methods, such as coagulation, membrane filtration, ion exchange, chemical precipitation, advanced oxidation processes and reverse osmosis, are employed to remove heavy metal ions from wastewater [6]. While some of these methods are highly efficient, they often come at a high cost requiring expensive equipment or chemicals, and significant energy consumption, or result in the formation of toxic sludge that demands proper treatment [7,8]. Sorbents are simple, efficient and economical solutions for removing heavy metal ions from wastewater [9]. Activated carbon and ion-exchange resins are commonly used for the removal of heavy metal ions from wastewater but since they are expensive, much of the research is focused on the substitution of these pollutants by some low-cost alternatives. Various waste materials (wood, peanut pellets, hazelnut shell, corncob, lignin, sawdust, etc.) have been investigated as potential sorbents for heavy metal ions, and despite numerous advantages, their often lower sorption capacities compared to conventional sorbents, such as activated carbon, alumina, or zeolite, are found to be critical [5,9,10]. Natural textile fibers used as sorbents have many advantages including their biodegradability and abundance in pristine and waste forms [5,11]. One of the directions in textile waste recycling could be the production of fibrous sorbents. The fibrous structure of textile waste enables the fibers to be processed in a manner that produces a self-supporting fiber matrix in the form of non-woven sorbents (NWS). In this way, the manipulation of such sorbents is easier and additional pollution from loose fibers themselves is prevented.

**Figure 1:**
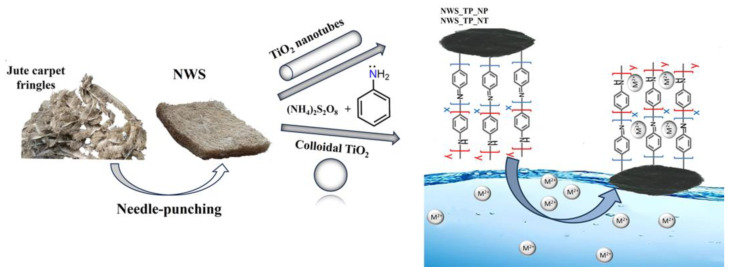
Scheme of NWS fabrication, coating with the PANI/TiO_2_ nanocomposite and proposed sorption mechanism.

In this study, a solution is proposed to address both issues: textile waste and the presence of heavy metal ions in water. The sorbent was derived from post-industrial textile waste obtained from a local carpet company. Discarded parts of the jute carpet fringes were utilized for the production of a non-woven material suitable for the sorption of heavy metal ions. NWS was manufactured by the needle punch process. In order to enhance its sorption properties for heavy metal ions, NWS was in situ modified with polyaniline (PANI) in the presence of TiO_2_ nanostructures (Figure 1). PANI is an environmentally friendly, low-cost polymer that is easy to synthesize [12,13,14]. Due to the numerous amino and imino groups present in its structure, PANI exhibits high sorption capacities for metal ions. The lone pair of electrons in the sp^3^ hybrid orbital of nitrogen can form a coordinate bond with metal ions [12]. To date, vast research has been focused on PANI as a sorbent for heavy metal ions due to simple synthesis routes, tunable morphology, the possibility of chemical regeneration by doping/de-doping, and cheap precursors [15], but PANI nanostructured sorbents are not convenient for practical use as they require additional separation from an aquatic medium. This drawback could be overcome by developing nanocomposites and utilizing its ability to be combined with a variety of polymers, textiles and fillers through in situ polymerization. Many reported studies related to PANI composite materials deal in particular with the removal of cadmium and chrome from water [12,15,16,17,18,19].

Although polymerization of aniline is possible without TiO_2_ nanostructures on textile fibers/surfaces [12], our previous research clearly indicated that small amounts of TiO_2_ nanoparticles (NPs) ensured controlled polymerization of aniline and considerably more uniform polymer cover of the fiber surface [20,21]. An additional advantage of nanostructured PANI is a large surface area that makes the sorption even more efficient. In this study, TiO_2_ was synthesized in the form of NPs and nanotubes (NTs) with an idea to examine the influence of its shape and size on the synthesis of PANI and, consequently, on the sorption properties of the modified NWS. The sorption behavior of both unmodified and modified sorbents for Pb^2+^ and Cu^2+^ ions was investigated in batch mode taking into consideration the influence of contact time, concentration, pH value, and temperature as well as the possibility of their recovery for repeated use.

## 2. Results and Discussion

### 2.1. Chemical, Morphological and Electrokinetic Characterization of Sorbents

**Figure 2:**
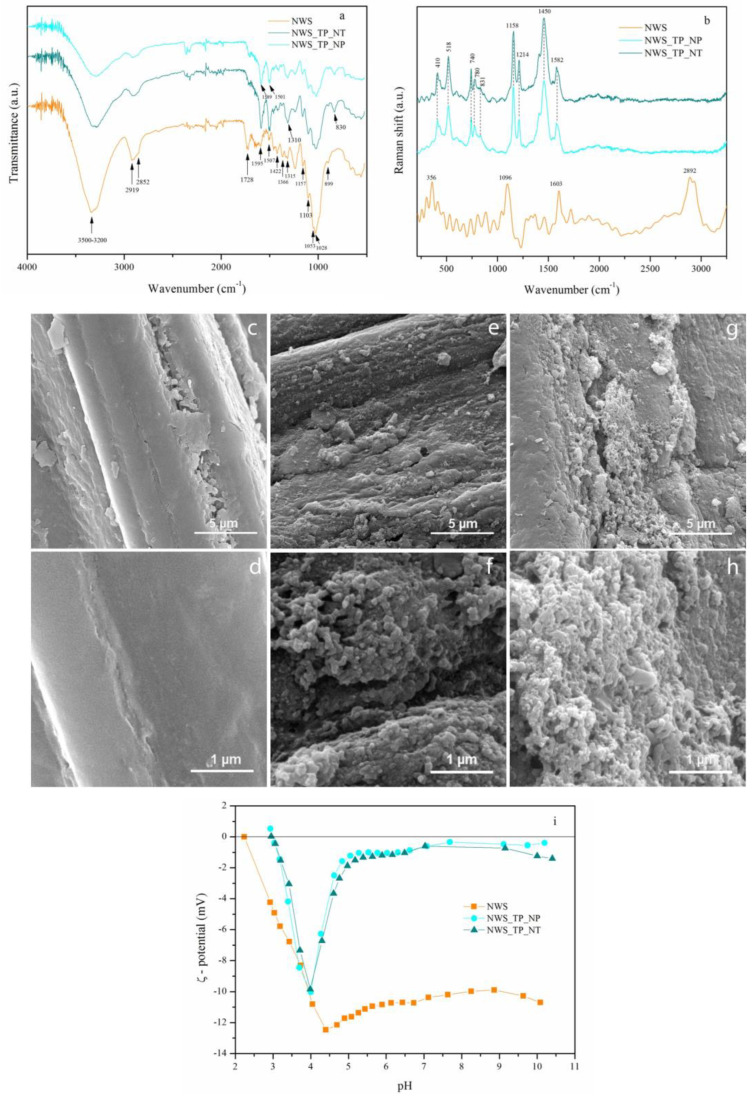
Chemistry, morphology and electrokinetic properties of control and modified NWSs: FTIR spectra of NWS, NWS_TP_NP, and NWS_TP_NT (**a**), Raman spectra of NWS, NWS_TP_NP, and NWS_TP_NT (**b**), FESEM micrographs of NWS (**c**,**d**), NWS_TP_NP (**e**,**f**), and NWS_TP_NT (**g**,**h**) under different magnifications (×10,000 and ×50,000), electrokinetic properties of NWS, NWS_TP_NP and NWS_TP_NT (**i**).

In order to analyze the chemical structure of the untreated and PANI/TiO_2_-treated sorbents, FTIR and Raman spectroscopies were applied. The FTIR spectra of non-woven sorbent (NWS), sorbent coated with PANI/TiO_2_ nanoparticles (NWS_TP_NP), and sorbent coated with PANI/TiO_2_ nanotubes (NWS_TP_NT) are presented in Figure 2a. The FTIR spectrum of NWS indicates a chemical structure typical for lignocellulose: a broad band in the range of 3500–3200 cm^−1^ (O–H stretching vibrations in cellulose and hemicellulose) [22]; a broad band with peaks at 2919 and 2852 cm^−1^ (asymmetric C–H stretching in aliphatic hydrocarbons and symmetric stretching, present in lignocellulosic constituents) [23]; the peak at 1728 cm^−1^ (C=O groups of acetyl ester in hemicellulose and aldehyde in lignin); peaks at 1595 and 1507 cm^−1^ (C=C vibrations of skeletal aromatic rings in lignin); the peak at 1422 cm^−1^ (skeletal aromatic vibration combined with the C-H in-plane deformation in -OCH_3_) [24]; bands at 1366 cm^−1^ (C–H bending (deformation stretch) vibrations) and at 1315 cm^−1^ (C–H wagging vibrations in cellulose) [25]; bands at 1157 and 1103 cm^−1^ (asymmetric bridge C–O–C), 1053 cm^−1^ (asymmetric in plane ring stretching), 1028 cm^−1^ (C–O stretching) and 899 cm^−1^ (asymmetric out-of-phase ring stretching at C_1_–O–C_4_ β-glucosidic bond) in cellulose [25,26].

The intensities of all the mentioned characteristic bands of lignocellulose decreased after modification with PANI/TiO_2_ nanocomposite. This is attributed to a complete cover of jute fibers with polymer material, which masked the lignocellulose groups. Additionally, new bands appeared at 1589 and 1501 cm^−1^ corresponding to the C=C stretching in quinoid and benzenoid ring stretching vibrations, respectively [27]. Nearly equal intensities of the bands at 1589 and 1501 cm^–1^ indicate the oxidation state of PANI, revealing its emeraldine base form, which comprises approximately equal amounts of benzenoid and quinonoid units [17,28,29]. The band at 1310 cm^–1^ represents C-N stretching of a secondary aromatic amine of PANI [27]. The peak at 830 cm^–1^ is ascribed to C−H bending out of the plane of para-substituted aromatic ring in PANI [27,28,29,30]. Comparison between the FTIR spectra of the NWS before and after modification confirms a successful in situ synthesis of PANI/TiO_2_ nanocomposite on the fiber surface. No significant differences between the spectra of NWS_TP_NP and NWS_TP_NT were found.

Similar conclusions were derived from the evaluation of the Raman spectra (Figure 2b). Again, the bands characteristic for lignocellulose fibers were clearly distinguished in NWS spectrum: the band at 2892 cm^−1^ (C–H stretching vibration of cellulose) [31,32]; the band at 1096 cm^−1^ (C–O–C asymmetric stretching vibrations of the glycosidic bond in cellulose) [31,32]; the band at approximately 356 cm^−1^ (β-D-glucosides bond in cellulose) [31,32]; and the band at 1603 cm^−1^ (C=C aromatic ring stretching vibration in lignin) [31,32,33]. The Raman spectra of NWS_TP_NP and NWS_TP_NT both display bands at 1582 cm^−1^ corresponding to C=C and C∼C (‘∼’ indicates the bond intermediate between the single and double bond) stretching vibrations of the semiquinonoid (SQ) and quinonoid (Q) rings of PANI, respectively [34,35]. The benzenoide (B)-substituted aromatic ring C=C and C–N stretching vibrations are found at 1445 cm^−1^ and 1214 cm^−1^, respectively [34,35]. A band that appears at 1158 cm^−1^ is associated with the Q ring’s C–H in-plane bending vibration [34,35]. Further, the bands at 831 and 780 cm^−1^ are ascribed to wagging vibration and C–H deformation inside the Q ring, respectively [34,35]. The vibrational bands at lower wavenumbers, 518 and 410 cm^–1^ are associated with B ring deformation vibration and C–N–C torsion vibration, respectively [34,35]. All bands detected in both NWS_TP_NP and NWS_TP_NT correspond to the half oxidized form of PANI, i.e., emeraldine base form. An absence of the bands originating from cellulose and lignin in these samples is in line with FTIR results (Figure 2) and indicates that the nanocomposite material has completely covered the surface of NWS. This assumption was confirmed by FESEM analysis.

FESEM images of NWS, NWS_TP_NP and NWS_TP_NT under different magnifications (×10,000 and ×50,000) are shown in Figure 2c–h. A characteristic coarse multicellular jute fiber with lignin playing a key role in connecting the elementary jute fibers is evident in the FESEM image of NWS (Figure 2c,d) [36]. Our previous research emphasized the significance of TiO_2_ NPs in synthesizing PANI to achieve complete and uniform cover of PET or cotton fibers [20,30]. Particularly the images taken at higher magnifications (Figure 2f,h) prove that jute fibers in NWS_TP_NP and NWS_TP_NT are completely covered with PANI nanocomposite, though the thickness of the nanocomposite layer varies over the fiber surface. No significant difference in the surface morphology of fibers between NWS_TP_NP and NWS_TP_NT was observed. A specific network structure of the ribbon-like nanostructure of PANI/TiO_2_ can be noticed, as already described in the literature [30,37].

In addition to morphological and chemical changes, the coating of NWS with PANI/TiO_2_ nanocomposite caused the changes in electrokinetic properties. These changes were evaluated by ζ-potential measurements performed at pH values ranging from 3 to 10. Although ζ-potential essentially explains the solid surface charge, it can also be considered as a subtle indicator of the fiber surface modifications [38]. It is strongly affected by the chemical composition, polarity, porosity, specific surface of fiber and fiber swelling in an aqueous medium [39]. The dependence of ζ-potential vs. pH for the NWS, NWS_TP_NP and NWS_TP_NT is shown in Figure 2i.

All samples are negatively charged in the whole range of investigated pH values. A negative ζ-potential plateau value of NWS in the alkaline region is characteristic for jute fibers and it is attributed to the dissociation of the present hydroxyl and carboxyl groups [38,39,40]. Hydroxyl groups of cellulose and some functionalities in lignin are considered as weak acids, while uronic acids in hemicellulose behave as stronger acids [38]. The negative ζ-potential plateau values of NWS_TP_NP and NWS_TP_NT considerably decrease compared to NWS due to the presence of PANI. However, the negative ζ-potential of these samples drops sharply at ~ pH 5 that is attributed to fiber swelling. Namely, the ζ-potential of any fiber decreases with increasing adsorption of water and swelling, i.e., a competitive adsorption of water molecules significantly influences the amount of adsorbed potential-determining ions of electrolyte, which decreases during the swelling process, leading to a simultaneous decrease in the ζ-potential [39]. An increase in ζ_min_/ζ_plateau_ from 1.2 in case of the NWS to 27 and 16 for NWS_TP_NP and NWS_TP_NT, respectively, suggests increased swelling of modified fibers [41]. The isoelectric points of NWS, NWS_TP_NP and NWS_TP_NT are 2.24, 2.99 and 2.96, respectively. Obviously, the isoelectric points of modified samples are shifted towards higher pH values. The negative charge on the surface of these samples beyond the isoelectric point occurs because of the deprotonation of amine groups of PANI [42]. NWS_TP_NP and NWS_TP_NT show almost identical electrokinetic properties.

### 2.2. The Influence of Contact Time on the Sorption of Metal Ions

**Figure 3:**
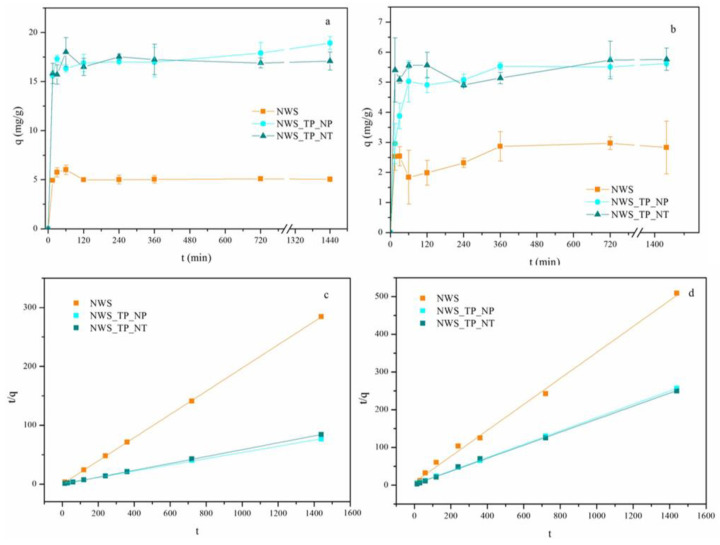
Adsorption kinetics of (**a**) Pb^2+^ ions, (**b**) Cu^2+^ ions (C_0_ = 500 mg/L, 20 °C, pH 5.0), and pseudo-second-order plots for the sorption of (**c**) Pb^2+^ ions and (**d**) Cu^2+^ ions by NWS, NWS_TP_NP, and NWS_TP_NT.

The influence of contact time on the uptake of Pb^2+^ and Cu^2+^ ions by NWS, NWS_TP_NP and NWS_TP_NT is shown in Figure 3. After the fast uptake of both metal ions in the first hour, the sorption slowed down until the equilibrium was reached, independently of the investigated sorbent. In the initial stage of the sorption process, the available sites in the sorbents are rapidly occupied by metal ions due to the concentration gradient between the liquid and solid phases. Later, their number declined because of the lack of vacant sites in the sorbent and the decreased concentration of metal ions in the solution [16,43]. The equilibrium was achieved after approximately 2 h for NWS in the case of the Pb^2+^ ions, while it took 6 h for all other samples for both Pb^2+^ and Cu^2+^ ions.

The sorption capacities of NWS, NWS_TP_NP, and NWS_TP_NT for Pb^2+^ ions after 24 h were 5.06, 18.94, and 17.11, respectively. Obviously, the modification of NWS with PANI/TiO_2_ nanocomposite resulted in approximately 3.5-fold larger sorption capacities for Pb^2+^ ions. The sorption capacity for Cu^2+^ ions was significantly smaller: 2.83, 5.62, and 5.76 for NWS, NWS_TP_NP, and NWS_TP_NT, respectively. This time, the sorption capacities were doubled after in situ synthesis of PANI/TiO_2_ nanocomposite. No significant difference in sorption capacities and the trend of sorption between NWS_TP_NP, and NWS_TP_NT were noticed. A larger sorption capacity towards Pb^2+^ ions in comparison with Cu^2+^ ions is often ascribed to characteristics of the metal ions, such as ionic radii and Pauling electronegativity, but contradictory findings imply that the sorption mechanism is affected by nature of both adsorbent and adsorbate [29].

Although it is difficult to compare the obtained maximum sorption capacities with those in the literature as the experiments are performed under different conditions, the sorption capacities of several similar sorbents for Pb^2+^ and/or Cu^2+^ ions are given in Table 1.

Adsorption kinetics studies were performed using pseudo-first- and pseudo-second-order models. The experimental data showed very poor compliance with the pseudo-first-order model (Figure S1). The data in Table 2, Figure 3c,d indicate that pseudo-second-order kinetic model fits the sorption kinetics of Pb^2+^ and Cu^2+^ ions well, which is in good correlation with studies obtained with similar sorbents [12,14]. In addition, many sorption processes for the removal of metal ions from water by sorbents functionalized with PANI obey the pseudo-second-order reaction model [14]. The correlation coefficients R^2^ for the pseudo-second-order kinetic model exceed 0.99 for all investigated samples, and the calculated *q_e_* values are in alignment with the experimental data. This agreement suggests that the pseudo-second-order kinetic model describes the adsorption behavior well, implying that the chemisorption is the rate-controlling step in the adsorption of Pb^2+^ and Cu^2+^ ions with all tested samples [10].

### 2.3. Adsorption Isotherms

The Langmuir isotherm assumes monolayer adsorption and describes specific homogeneous adsorption sites on the surface of the sorbent. Once a site on the surface is occupied, no additional adsorption can take place at that certain site. On the other hand, the Freundlich isotherm model allows multilayer adsorption. Freundlich and Langmuir adsorption isotherms were used to describe the adsorption properties of NWS, NWS_TP_NP and NWS_TP_NT at equilibrium conditions. To this end, batch sorption experiments were carried out, where the sorption capacities of the samples at equilibrium (*q_e_*) and equilibrium concentrations of metal ions in the solution (*C_e_*) were determined. The linear forms of the Langmuir isotherms are presented in Figure S2, and the corresponding parameters are listed in Table 3. The Freundlich model gave a relatively poor fit to obtained isotherms of the control and modified samples. The Freundlich isotherms with corresponding data are shown in Figure S3 and Table S1 (see Supplementary Materials). The correlation coefficients (R^2^) in Table 3 suggest a better fit of the experimental data to the Langmuir isotherm. An excellent fit was obtained in case of the NWS_TP_NP and NWS_TP_NT for the sorption of both Pb^2+^ and Cu^2+^ ions. However, either Langmuir or Freundlich model does not describe the sorption behavior of NWS well for the removal of Cu^2+^ ions. 

### 2.4. The Influence of Initial Concentration on the Sorption of Metal Ions

**Figure 4:**
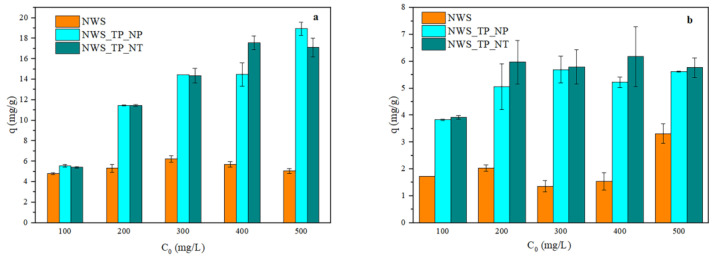
The influence of the initial concentration of metal ions on the sorption capacity of NWS, NWS_TP_NP, and NWS_TP_NT: (**a**) Pb^2+^ ions and (**b**) Cu^2+^ ions (pH 5.0, 20 °C, 24 h).

The influence of the initial concentration of metal ions on the sorption capacity is presented in Figure 4. Obviously, the sorption capacity of NWS was not affected by the initial concentration of Pb^2+^ ions (Figure 4a). A similar trend occurred with Cu^2+^ ions (Figure 4b), though the uptake slightly increased for the initial concentration of 500 mg/L. Figure 4a also shows that the higher the initial concentration of Pb^2+^ ions, the larger the sorption capacity of NWS_TP_NP and NWS_TP_NT, but no significant difference between these samples is noticed. The influence of the initial concentration of Cu^2+^ ions on the sorption capacity of NWS_TP_NP and NWS_TP_NT was less prominent particularly in the range of concentrations between 200 and 500 mg/L. Similar behavior was reported with recycled fibers (78% PET, 20% cotton, 2% others) in the work reported by Tan et al. [58].

### 2.5. The Influence of pH on the Sorption of Metal Ions 

**Figure 5:**
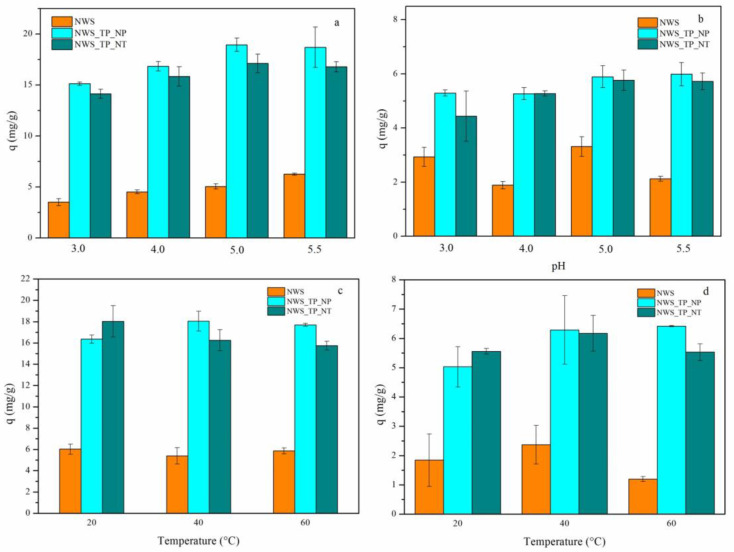
The influence of pH on the sorption of (**a**) Pb^2+^ ions and (**b**) Cu^2+^ ions (*C*_0_ = 500 mg/L, 20 °C, 24 h) and influence of temperature on the sorption of (**c**) Pb^2+^ ions and (**d**) Cu^2+^ ions (*C*_0_ = 500 mg/L, pH 5.0, 60 min) by NWS, NWS_TP_NP, and NWS_TP_NT.

The influence of pH on the sorption of Pb^2+^ and Cu^2+^ ions was studied in the pH range where the possibility of metal hydroxides precipitation was excluded. Since the precipitation of Cu(OH)_2_ begins already at ~pH 6, the sorption behavior was accessed at pH 3.0, 4.0, 5.0 and 5.5. The pH values below 3 were also eliminated because the jute fibers could be damaged, potentially affecting the reliability of conclusions. The effect of pH on the sorption capacity of NWS, NWS_TP_NP and NWS_TP_NT for Pb^2+^ and Cu^2+^ ions is shown in Figure 5a,b, respectively. A small increase in sorption capacity with an increase in pH was observed primarily in the case of Pb^2+^ ions. The sorption capacities of NWS_TP_NP were slightly larger compared to NWS_TP_NT. In contrast, a negligible difference in sorption capacities between these samples was found at higher pH values when Cu^2+^ ions were sorbed. In the case of Cu^2+^ ions, a specific trend could not be established for NWS. No significant difference in the sorption capacity of modified samples was evident at pH 5.0 and 5.5 independently of metal ion. Thus, the largest sorption capacities are achieved at pH 5.0–5.5, which is in line with findings reported by Huang et al. during the sorption of Cd^2+^ ions by jute fibers functionalized with PANI [14]. They explained this observation by the coordinative binding of Cd^2+^ ions to an electron pair on the imine nitrogen atoms in the emeraldine base form of PANI, where the resulting quinone segments could rearrange and form more stable semiquinone structures. Namely, unlike NWS, where the metal ion uptake is mostly governed by coordinative binding between phenolic groups of lignin in jute and metal ions, PANI in NWS_TP_NP and NWS_TP_NT can bind metal ions through a coordinative binding between positively charged metal ions and free electrons in the sp^3^ orbital of nitrogen from imino or amino group of PANI [12]. Ion exchange is also reported as one of the mechanisms involved in biosorption processes [59].

The trend of sorption capacity decline with the decrease in pH is explained in the literature by the competition between protons and metal ions during binding to the sorbent surface as the medium becomes more acidic as well as by repulsion between metal ions and positively charged sorbent surface [60]. As the pH increases, the deprotonation of amino and imino groups takes place, leading to enhanced sorption of metal ions. The results from Figure 5a,b clearly confirm that the process of deprotonation has already started at pH 3.0 as the isoelectric points of all samples were lower than pH 3.0.

### 2.6. The Influence of Temperature on the Sorption of Metal Ions

The temperature did not significantly affect the sorption of both metal ions, as can be seen in Figure 5c,d. Different behavior was noticed in the sorption of Pb^2+^ and Cu^2+^ ions by NWS. The uptake of Pb^2+^ ions seem to be independent of temperature while in the case of Cu^2+^ ions, after a slight increase at 40 °C, the uptake decreased at 60 °C. The decrease in metal ion uptake with temperature was detected in similar lignocellulose fibers and it is assumed that sorption is an exothermic process. A small increase in the uptake of both metal ions with elevation of temperature was observed in the case of NWS_TP_NP, indicating that sorption is an endothermic process. No specific trend was established for NWS_TP_NT.

### 2.7. Reusability of Sorbents

**Figure 6:**
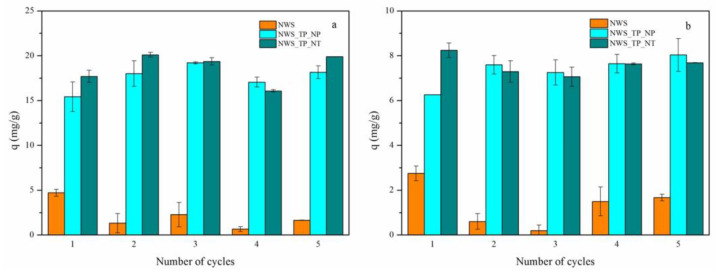
Reusability of NWS, NWS_TP_NP, and NWS_TP_NT for the sorption of (**a**) Pb^2+^ ions and (**b**) Cu^2+^ ions (*C*_0_ = 500 mg/L, 20 °C, pH 5.0, 24 h).

A sustainable sorbent for the removal of heavy metal ions should be easily regenerated and it should provide reuse, minimizing the waste after exploitation and lowering the overall costs of product. This study introduces a well-optimized desorption procedure designed to completely remove adsorbed metal ions from the saturated sorbent. The desorption process was performed in an acidic medium with 0.1 M HNO_3_. The presence of a high concentration of H^+^ ions from HNO_3_ stimulates the competition with metal ions. H^+^ ions occupy the sites on the sorbent where metal ions were bound, initiating their release into the acid solution [14,61]. Both the concentration of desorbed metal ions after each cycle and the sorption capacity for each cycle were monitored. Sorption capacities are shown in Figure 6. A reduction in sorption capacities is evident in NWS samples: 35% of the initial capacity for Pb^2+^ ions and 60% for Cu^2+^ ions were reached after 5 desorption/sorption cycles.

In contrast, the sorption capacities of NWS_TP_NP for both metal ions were slightly larger after 5 desorption/sorption cycles. In the case of NWS_TP_NT, a decrease in sorption capacity by approximately 10% was detected when Cu^2+^ ions were sorbed. Generally, a positive outcome in the reuse of PANI/TiO_2_-treated sorbents can be primarily attributed to the specific properties of PANI. Through de-doping in 0.5 M NaOH to form the emeraldine base, PANI becomes equally or even more enriched in available amino and imino groups at the surface. Additionally, as demonstrated by Shukla and Pai (2005) [62], neutralization with NaOH after the desorption of metal ions in acid is essential to restore the initial adsorption capacity. The retention of H^+^ ions on the sorbent could otherwise reduce the pH value of the metal ions solution in the new cycle and thereby decrease the maximum sorption capacity [62].

## 3. Materials and Methods

### 3.1. Materials

Post-industrial waste in the form of jute carpet fringes with small polyamide (PA) patches was supplied from a local carpet company in Serbia. A fibrous web was formed from garneted fringes and needle punched in the local company “Meteks” (Mladenovac, Serbia). The area density of the produced NWS is 530 g/m^2^. The thickness of the NWS measured using a vernier caliper is 5.6 ± 0.3 mm. The bursting strength determined according to the standard SRPS F.S2.022/1: 2018 [63]—Textiles—Determination of the fabric resistance against busting is 287.8 ± 27 N. Quantitative chemical analysis according to ISO 1833-11: 2017 [64]—Textiles—Quantitative chemical analysis—Part 11: Mixtures of certain cellulose fibres with certain other fibres (method using sulfuric acid) indicated that the NWS comprises 90% jute and 10% PA fibers.

### 3.2. In Situ Polymerization of PANI in the Presence of TiO_2_ NPs/NTs

An amount of 1.00 g of NWS was immersed in a colloid of TiO_2_ NPs (5 mL of 0.2 M TiO_2_ colloid was poured in 45 mL of water) or in a suspension of TiO_2_ NTs (80 mg TiO_2_ NTs was dispersed in 50 mL of deionized water in an ultrasonic bath for 30 min). The 0.2 M colloid of TiO_2_ NPs was synthesized by acid hydrolysis of TiCl_4_ (Sigma-Aldrich, France) as described in our previous report [65]. TiO_2_ NTs were synthesized by a hydrothermal method using a commercial TiO_2_ anatase powder (Fluka AG, Switzerland) as a precursor [66,67]. The soaked sample was stirred using a magnetic stirrer for 15 min and solutions of aniline (ANI, Acros Organic, Portugal) and ammonium peroxydisulfate (APS, TTT, Croatia) were simultaneously introduced when the in situ polymerization of ANI started. An aqueous solution of APS was obtained by dissolving 2.2820 g of APS in an appropriate volume of 0.5 M HCl (volumetric flask filled up to 25 mL). The concentration of the obtained solution was 0.4 mol/L. A solution of ANI was prepared by dispersing 0.73 g of ANI in an appropriate volume of 0.5 M HCl (volumetric flask filled up to 25 mL). The concentration of the solution was 0.32 mol/L. The polymerization process lasted 90 min. Afterward, the sample was washed with 1.25 L of deionized water. The process of PANI layer de-doping was conducted by treating the NWS with 50 mL of 0.5 M NaOH solution for 10 min. The de-doped sample was washed with 1 L of deionized water and dried at 40 °C for 24 h. The samples treated with PANI in the presence of TiO_2_ NPs and NTs are denoted NWS_TP_NP and NWS_TP_NT, respectively.

### 3.3. Characterization of Sorbents

The morphological properties of NWS, NWS_TP_NP, and NWS_TP_NT were analyzed by a field-emission scanning electron microscope (FESEM, Mira3 Tescan, Brno-Kohoutovice, Czech Republic). The samples were sputter coated with a thin layer of Au before the analysis.

The Raman spectra of the samples were recorded after excitation by a HeNe gas laser (at an excitation wavelength of 780 nm) and collected on a Thermo Scientific DXR Raman microscope, equipped with a research optical microscope and a CCD detector. The laser beam was focused on the sample placed on an X-Y motorized sample stage using an objective magnification of 50×. The scattered light was analyzed by the spectrograph with a 600 lines-per-mm grating. Laser power at the sample was 24 mW.

FTIR (Fourier Transform Infrared Spectroscopy) analysis was performed in the attenuated total reflectance (ATR) mode using a Nicolet iS5 FTIR spectrometer (Thermo Fisher Scientific) at 4 cm^−1^ resolution, over a range of 500–4000 cm^−1^.

ζ-potential was measured by the streaming potential method using a SurPASS Electrokinetic Analyzer (Anton Paar GmBH, Graz, Austria). Circular samples of NWS, NWS_TP_NP, and NWS_TP_NT with a diameter of 13 mm were left to swell in deionized water for 24 h prior to measurement.

### 3.4. Evaluation of NWS, NWS_TP_NP, and NWS_TP_NT Sorption Performance

Stock solutions of Pb^2+^ and Cu^2+^ ions (1000 mg/L) were prepared by dissolving Pb(NO_3_)_2_ (Centrohem, Serbia) and Cu(NO_3_)_2_·2 H_2_O (Fisher Chemicals, USA) in distilled water. These was further diluted to obtain desired concentrations. All experiments were performed in batch mode.

The influence of contact time on the uptake of Pb^2+^ and Cu^2+^ ions was examined in precisely defined time intervals. An amount of 1.00 g of sorbent was immersed in 50 mL of a metal ions solution (*C*_0_ = 500 mg/L) at pH 5.0. The Erlenmeyer flask with a sorbent was capped and placed in a laboratory shaker at 20 °C. The solution was shaken at 120 strokes/min. After 15, 30, 60, 120, 240, 360, 720 and 1440 min, a sample of the solution was taken and the concentration of heavy metal ions was measured by atomic absorption spectroscopy (AAS, Spectra AA 55B, Varian, Palo Alto, CA, USA). The metal ion uptake by the sorbents (*q*, mg/g) is calculated according to Equation:

(1) $$q=\frac{\left(C_{0}-C_{t}\right)\cdot V}{m}$$

where *C*_0_ is the initial concentration of metal ions solution (mg/L), *C_t_* is the concentration of metal ions solution after the sorption in defined time interval (mg/L), *V* is the volume of the metal ions solution (L), and m is the sorbent mass (g).

The described procedure was also applied for the evaluation of the influence of temperature on metal ion uptake, but the sorption process was carried out at 20 °C, 40 °C and 60 °C for 60 min. The influence of pH was examined in the same way at 20 °C and pH 3.0, 4.0, 5.0 and 5.5 for 24 h. The pH was adjusted with 0.1 M HNO_3_ (Zorka Pharma, Serbia) or 0.1 M NaOH (Lachema, Czech Republic) solutions. The experiments related to the influence of the initial concentration of metal ions were carried out in the same manner at 20 °C and pH 5.0 for 24 h. The initial concentrations were 100, 200, 300, 400 and 500 mg/L.

The possibility to recover the sorbent was tested in five cycles, each consisting of metal ions sorption and desorption phases. The sorption phase was performed in the same manner as previous experiments under the following conditions: *C*_0_ = 500 mg/L, pH 5.0 at 20 °C for 24 h. The ions desorption phase began with rinsing of the sample with sorbed ions with distilled water and subsequent drying in an oven at 60 °C. A dry sample was further immersed in 100 mL of 0.1 M HNO_3_ and shaken (120 strokes/min) at 20 °C. After 60 min, the sample was thoroughly rinsed with distilled water. NWS_TP_NP and NWS_TP_NT were immersed in 0.5 M NaOH (de-doping of the PANI layer) and stirred. After 10 min of stirring by magnetic stirrer at a speed of 200 rpm, the samples were rinsed with distilled water and dried at 60 °C. Dry samples were placed into the newly prepared metal ions solution when the next cycle started.

### 3.5. Mathematical Models

The pseudo-first- and pseudo-second-order models were used to evaluate the sorption kinetics of Pb^2+^ and Cu^2+^ ions. The linear forms of these models are defined by Equations (2) and (3), respectively:

(2) $$log\left(q_{e}-q_{t}\right)=logq_{e}-k_{1}t$$

(3) $$\frac{t}{q_{t}}=\frac{1}{k_{2}q_{e}^{2}}+\frac{t}{q_{e}}$$

where *q*_e_ (mg/g) is the sorption capacity at equilibrium, *q_t_* (mg/g) is the sorption capacity at time *t* (min), *k*_1_ (1/min) is the first-order rate constant and *k*_2_ (g/mg·min) is the second-order rate constant.

Langmuir and Freundlich isotherms as the most widely applied models for the description of sorption of metal ions from water were chosen for evaluation of sorption behavior in this study. Linear forms of these models are expressed in Equations (4) and (5), respectively:

(4) $$\frac{C_{e}}{q_{e}}=\frac{C_{e}}{q_{max}}+\frac{1}{K_{L}\cdot q_{max}}$$

(5) $$logq_{e}=\frac{1}{n}logC_{e}+logK_{F}$$

where *C_e_* (mg/L) is the equilibrium concentration of the metal ions solution, *q_e_* (mg/g) is the sorption capacity at equilibrium, *q_max_* is the maximum sorbent capacity (mg/g), *K_L_* is the Langmuir energy constant of adsorption, *K_F_* is the Freundlich capacity factor, and *n* is the Freundlich intensity factor.

## 4. Conclusions

The sorption capacity of cheap and biodegradable non-woven sorbent based on recycled jute for Pb^2+^ and Cu^2+^ ions could be enhanced by modification with PANI/TiO_2_ nanocomposite. FTIR and Raman spectroscopy revealed the formation of the emeraldine base form of PANI while FESEM analysis confirmed the formation of uniform cover consisting of a characteristic network structure of ribbon-like nanostructure of PANI/TiO_2_ all over the jute fibers. The shape of TiO_2_ nanostructures (nanoparticles and nanotubes) neither affected the morphology and chemistry of in situ synthetized nanocomposite nor the sorption behavior, proving their exclusive role in providing a uniform polymer coating. Electrokinetic measurements indicated that modification of material with PANI/TiO_2_ caused an increased swelling of fibers and a shift in isoelectric point towards higher values. The deprotonation of the amino and imino groups of PANI occurring at a pH above the isoelectric point of modified samples (pH 2.99 and 2.96 for the materials modified with TiO_2_ nanoparticles and nanotubes, respectively) led to enhanced sorption of both metal ions in the pH range between 3.0 and 5.5. The experimental data showed very good compliance with the pseudo-second-order kinetic model, which is characteristic for many biosorbents. Experimental data were best fitted for the Langmuir isotherm. The sorption capacity of both control and modified sorbents for Pb^2+^ ions was larger compared to Cu^2+^ ions. The modification with PANI/TiO_2_ nanocomposite brought about a more than 3-fold greater uptake of Pb^2+^ ions, and a 2-fold greater uptake of Cu^2+^ ions, independently of applied TiO_2_ nanostructure. The sorption was not significantly affected by temperature. Modified sorbents could be regenerated and reused at least 5 times with a negligible decrease in sorption capacity. This positive outcome is ascribed to the specific properties of PANI because de-doping in 0.5 M NaOH makes the PANI equally or even more augmented with available amino and imino groups at the surface. The obtained results indicate that adequately modified non-woven material based on recycled jute fibers could be utilized as an efficient sorbent for the removal of metal ions from water. Keeping in mind that recycling is one of the pillars of the circular economy model, this study shows that textile waste could be exploited as a useful resource for the production of sorbents. Future research steps should be directed towards further improvement of sorption capacity by activation and carbonization without deterioration of the non-woven structure.

## Figures and Tables

**Table 1:** Sorption capacities of similar sorbents for removal of Pb^2+^ and/or Cu^2+^ ions.

Sorbent	Sorption Capacity (mg/g)		Reference
	Pb^2+^	Cu^2+^	
Banana peel	2.18	Not studied	[44]
Beech sawdust	Not studied	4.5	[45]
Coconut tree seadust	25	3.9	[46]
Peanut shells	39	Not studied	[47]
Sweet poatato peel	18	Not studied	[48]
Flex fiber	10.7	9.9	[49]
Flex processing waste	13.4	8.3	[50]
Alfa grass fibers	14.49	11.90	[51]
Dried orange peel	Not studied	2.78	[52]
Avocado peel	4.93	Not studied	[53]
*L. nigrescens*	Not studied	12.15	[54]
Seaweed residue		9.09	
Washed pine wood fibers	Not studied	2.06	[55]
Pine wood fibers activated by sodium carbonate		4.26	
*Prosopis cineraria* leaf powder	10.238	Not studied	[56]
Waste yeastbiomass	Not studied	4.24	[57]
NWS	5.06	2.83	Current study
NWS_TP_NP	18.94	5.62	
NWS_TP_NT	17.11	5.77	

**Table 2:** Pseudo-second-order kinetic parameters.

Sample	Pb^2+^				Cu^2+^			
	*q_exp_*(mg/g)	*q_e_*(mg/g)	*k*_2_(g/mg·min)	R^2^	*q_exp_*(mg/g)	*q_e_*(mg/g)	*k*_2_(g/mg·min)	R^2^
NWS	5.06	5.07	0.22	0.99998	2.83	2.91	0.015	0.99675
NWS_TP_NP	18.94	18.92	0.003	0.9988	5.62	5.67	0.011	0.99982
NWS_TP_NT	17.11	17.08	0.62	0.99991	5.77	5.79	0.012	0.99856

**Table 3:** Regression data of the Langmuir isotherm.

Sample	Pb^2+^			Cu^2+^		
	*q_max_*(mg/g)	*K_L_*(L/g)	R^2^	*q_max_*(mg/g)	*K_L_*(L/g)	R^2^
NWS	5.823	0.520	0.993	2.593	0.0123	0.548
NWS_TP_NP	14.503	4.389	0.999	5.555	0.290	0.996
NWS_TP_NT	17.976	0.352	0.997	6.139	0.399	0.999

## Data Availability

Data are contained within this article.

## Supplementary Materials

The following supporting information can be downloaded at: https://www.mdpi.com/article/10.3390/molecules29184366/s1, Figure S1: Pseudo-first order plots for the sorption of (a) Pb^2+^ ions, and (b) Cu^2+^ ions by NWS, NWS_TP_NP, and NWS_TP_NT; Figure S2. Langmuir adsorption isotherms for removal of (a) Pb^2+^ ions, and (b) Cu^2+^ ions (pH 5.0, 20 °C, 24 h); Figure S3. Freundlich adsorption isotherms for removal of (a) Pb^2+^ ions, and (b) Cu^2+^ ions (pH 5.0, 20 °C, 24 h), Table S1: Regression data of Freundlich isotherm.
