# Supplementary material for: Recycled Jute Non-Woven Material Coated with Polyaniline/TiO2 Nanocomposite for Removal of Heavy Metal Ions from Water
Source: Molecules. 2024 Sep 14;29(18):4366. doi: 10.3390/molecules29184366 (PMC11434075; doi:10.3390/molecules29184366)
Supplement: Supplementary file 1 [file molecules-29-04366-s001.zip › molecules-3184721-supplementary.pdf]

Supplementary material

Recycled jute non-woven material – A low-cost biosorbent for removal of heavy metal ions from water

Aleksandar Kovačević[1], Marija Radoičić[2], Darka Marković[3], Zoran Šaponjić[4], Maja Radetić[1]

[1]*Faculty of Technology and Metallurgy, University of Belgrade, 11000 Belgrade, Serbia*

[2]*"Vinča" Institute of Nuclear Sciences, University of Belgrade, 11000 Belgrade, Serbia*

[3]*Innovation Centre of the Faculty of Technology and Metallurgy, University of Belgrade, 11000 Belgrade, Serbia*

[4]*Institute of General and Physical Chemistry, University of Belgrade, 11000 Belgrade, Serbia*

**Correspondence: maja@tmf.bg.ac.rs*

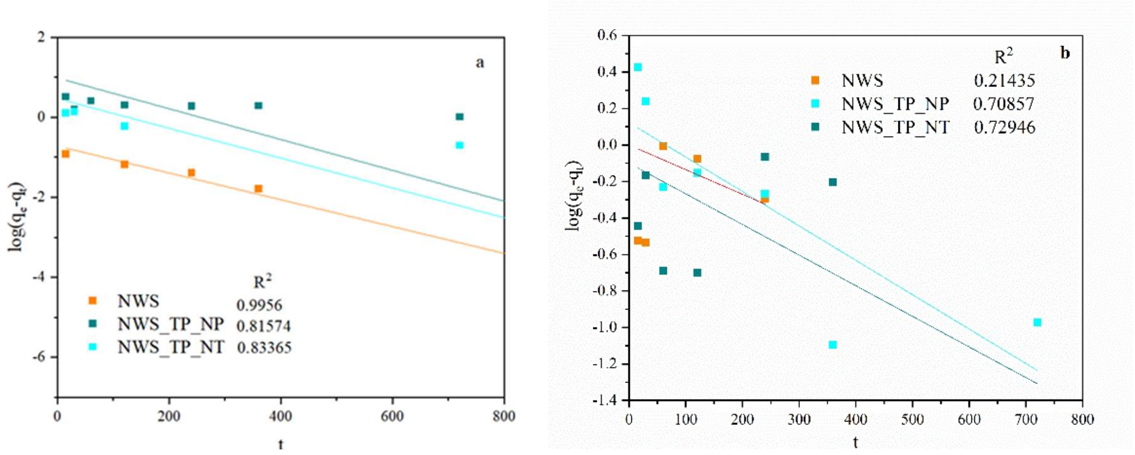

Figure S1. Pseudo-first order plots for the sorption of (a) Pb^{2+} ions, and (b) Cu^{2+} ions by NWS, NWS_TP_NP, and NWS_TP_NT.

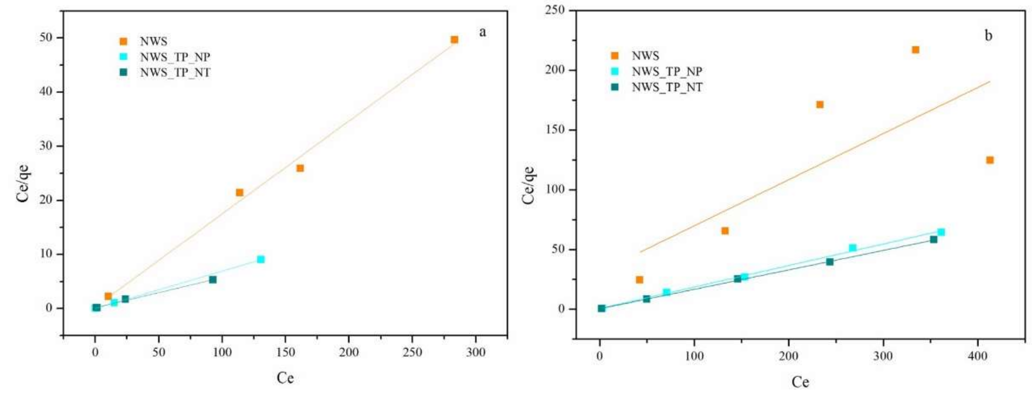

Figure S2. Langmuir adsorption isotherms for removal of (a) Pb^{2+} ions, and (b) Cu^{2+} ions (pH 5.0, 20°C, 24 h).

Table S1. Regression data of Freundlich isotherm

Sample	Pb^{2+}			Cu^{2+}		
	**n**	**K_F ((mg/g)·(L/mg)$^{1/n}$)**	**R^2**	**n**	**K_F ((mg/g)·(L/mg)$^{1/n}$)**	**R^2**
NWS	15.593	4.127	0.695	9.187	1.078	0.0823
NWS_TP_NP	7.442	8.765	0.714	12.713	3.572	0.914
NWS_TP_NT	4.842	7.304	0.748	11.494	3.832	0.902

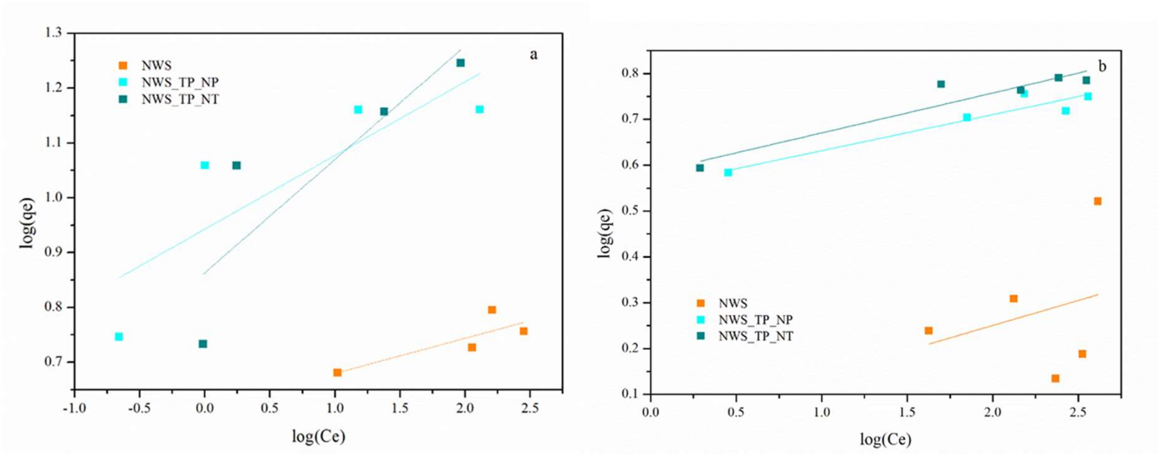

Figure S3. Freundlich adsorption isotherms for removal of (a) Pb^{2+} ions, and (b) Cu^{2+} ions (pH 5.0, 20°C, 24 h)
